# Efficacy of oral prolonged‐release mesalazine in moderately active ulcerative colitis

**DOI:** 10.1002/jgh3.12935

**Published:** 2023-07-07

**Authors:** Kristine Paridaens, John R Fullarton, Simon P L Travis

**Affiliations:** ^1^ Medical Affairs Ferring International Center St‐Prex Switzerland; ^2^ HEOR Violicom Medical Limited Aldermaston UK; ^3^ NIHR Oxford Biomedical Research Centre Oxford University Hospitals NHS Foundation Trust, John Radcliffe Hospital Oxford UK

**Keywords:** aminosalicylates, delayed‐release, effectiveness, inflammatory bowel disease, mesalamine

## Abstract

New meta‐analyses are presented that provide further evidence supporting the effectiveness of oral prolonged‐release mesalazine compared to other oral mesalazines as induction therapy in patients with moderately active ulcerative colitis.

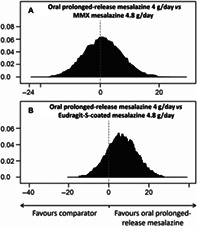

## Introduction

Mesalazine (5‐aminosalicylate, 5‐ASA) is the recommended first‐line treatment for mild to moderate ulcerative colitis (UC).^1^, ^2^ Oral prolonged‐release mesalazine (hereafter OPRM; Pentasa; Ferring Pharmaceuticals) is a time‐dependent release formulation of mesalazine, containing granules coated with cellulose polymers, and provides a continuous release of 5‐ASA throughout the gastrointestinal tract, independent of luminal pH and gut transit time.^3^ Commensurate with its indication, the major clinical evidence presented for OPRM in UC has tended to cover the spectrum of mild to moderate disease severity,^4^ with limited separate analyses of patients with moderate disease alone or head‐to‐head comparisons with other mesalazines. The analyses presented here provide further supporting evidence for the effectiveness of OPRM as induction therapy in patients with moderately active UC.

## Evidence

**Figure 1:**
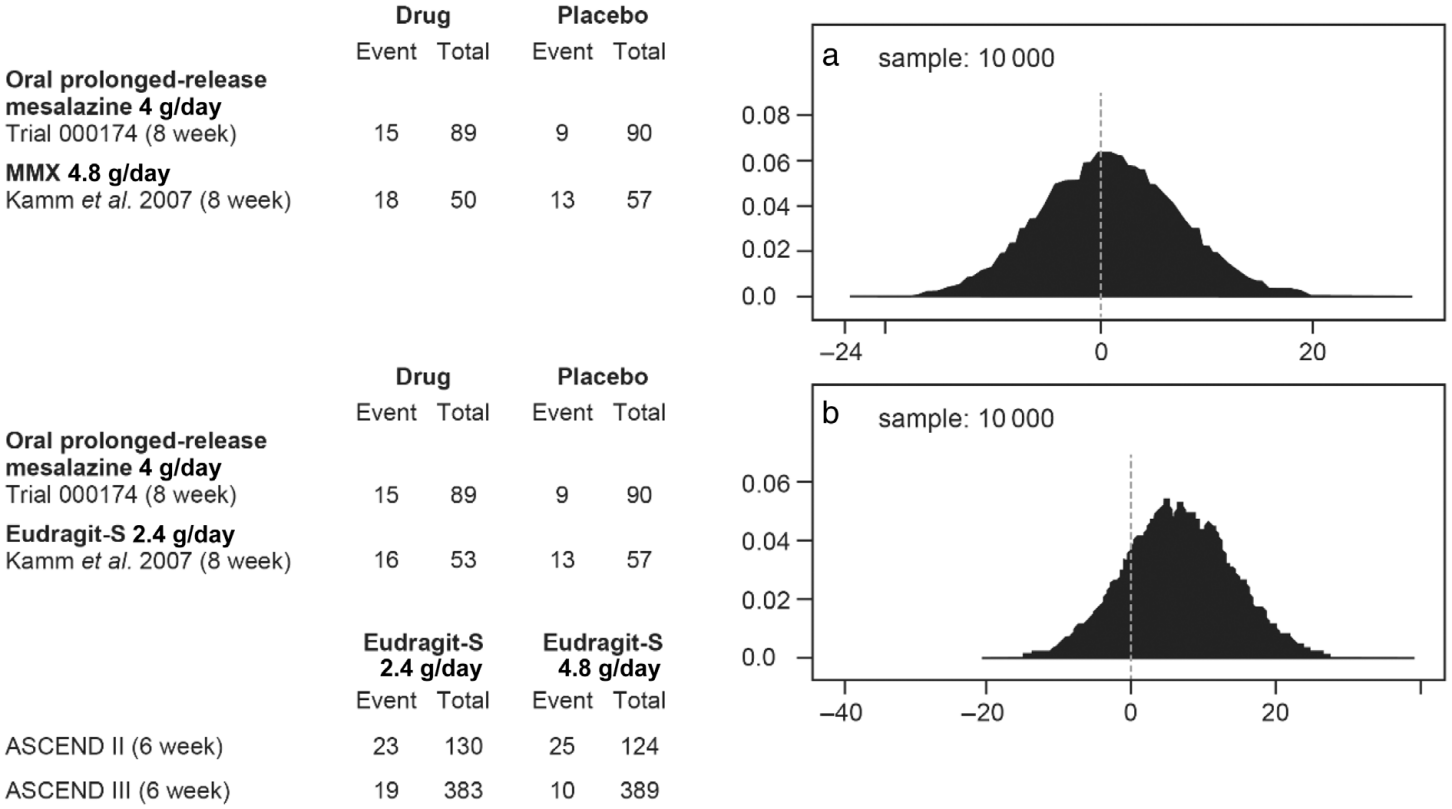
Oral prolonged‐release mesalazine 4 g/day *versus* MMX mesalazine 4.8 g/day (a) and Eudragit‐S‐coated mesalazine 4.8 g/day (b) for the induction of remission in patients with moderate ulcerative colitis.

Meta‐analyses were conducted to compare high‐dose (4 g) OPRM with other oral mesalazine formulations (4.8 g) using a Bayesian inferential aggregate of binomial stochastic distribution functions with 10 000 sampling iterations (WINBUGS 1.4.3, Imperial College and MRC, UK). The included studies were identified from two published systematic literature reviews: one focused specifically on OPRM, which included previously unpublished data^4^; and the other undertaken by Cochrane, which included all mesalazines for induction of remission in UC.^5^ Targeted literature searches were also carried out to identify any additional studies published subsequent to these two reviews. The first analysis compared OPRM 4 g/day (Trial 000174)^6^ and MMX mesalazine 4.8 g/day^7^ using the endpoint of induction of combined clinical and endoscopic remission, with placebo as the common comparator (link between OPRM and MMX mesalazine). In this analysis, no significant difference was found, with the peak of the difference distribution almost precisely coinciding with the zero point (representing the position where the treatment efficacies are equivalent; Fig. 1a). A second analysis compared OPRM 4 g/day (Trial 000174)^6^ with Eudragit‐S‐coated mesalazine (ESM hereafter) 4.8 g/day (ASCEND II^8^ and III^9^) for the achievement of composite remission, using ESM 2.4 g/day and placebo^7^ as the common comparators. Here, the zero point was within the 80% the credible interval, again indicating no significant difference between treatments (Fig. 1b). Limitations of the meta‐analyses included variation across studies in how moderate disease and remission were defined (Table 1). Bayesian meta‐analyses account for heterogeneity across studies by enabling prior probability component variables to comply with their unique distributions through Gibbs sampling (Markov chain Monte Carlo, MCMC) during the process of generating posterior probabilities.

**Table 1:** Definitions of remission from studies included in meta‐analyses of oral prolonged‐release mesalazine 4 g/day *versus* MMX mesalazine 4.8 g/day and Eudragit‐S‐coated mesalazine 4.8 g/day for the induction of remission in patients with moderate ulcerative colitis (UC)

Study	Treatment	Definition of remission
Trial 000174^6^	Oral prolonged‐release mesalazine 4 g/day, placebo	Clinical and endoscopic response score of rectal bleeding scores of 0 and stool frequency score of 0 or 1 with at least 1 point decrease from baseline, with an endoscopic score of 0 or 1. Moderate disease is defined as a modified Mayo score of 7–10
Kamm *et al*. 2007^7^	MMX 4.8 g/day, Eudragit‐S‐coated mesalazine 2.4 g/day, Placebo	Modified UC‐DAI score of ≤1 with a score of 0 for rectal bleeding and stool frequency and at least 1‐point reduction from baseline in sigmoidoscopy score. Moderate disease is defined as modified UC‐DAI score of 6–10
ASCEND II^8^	Eudragit‐S‐coated mesalazine 2.4 g/day, Eudragit‐S‐coated mesalazine 4.8 g/day	Complete remission defined as normal stool frequency, no rectal bleeding, patient's functional assessment score of 0, normal endoscopy findings, and PGA assessment score 0. Moderate disease is defined as a PGA score of 2
ASCEND III^9^	Eudragit‐S‐coated mesalazine 2.4 g/day, Eudragit‐S‐coated mesalazine 4.8 g/day	Complete remission defined as PGA score of 0 (i.e. complete resolution or normalization of stool frequency, rectal bleeding, and sigmoidoscopy with contact friability assessment score). Moderate disease is defined as PGA score of ≥1 point in both the stool frequency and rectal bleeding clinical assessments and a score of ≥2 points in the sigmoidoscopy assessment with a positive friability assessment

PGA, Physician's Global Assessment; UC‐DAI, UC Disease Activity Index.

Further evidence in moderate UC was derived from a double‐blinded, randomized controlled trial, which compared OPRM 2.25 g/day and ESM 2.4 g/day.^10^ In this study, there was no significant difference reported between the two mesalazine formulations for decrease in Ulcerative Colitis Disease Activity Index (UC‐DAI) scores (primary study outcome; difference between treatments: 0.1; 95% confidence interval [CI]: −1.3 to 1.6).^10^ A dose of 3.6 g/day ESM was found to be more efficacious than OPRM 2.25 g/day (*P* = 0.003), evidencing the increased efficacy with higher doses of mesalazine.^10^ Real‐world evidence on the use of OPRM was identified in the recently published QUARTZ study, which found no differences in outcomes (clinical remission, endoscopic activity, or relapse rates) between those with mild or moderate disease.^11^ This study also reported that OPRM significantly improved the mean Short Inflammatory Bowel Disease Questionnaire (SIBDQ) scores after 8 weeks′ treatment in patients with moderate disease (43.3 *vs* 35.9 at baseline; *P* < 0.001), which was maintained at month 12.^11^

As a proxy for greater disease severity, meta‐analyses were undertaken of data from patients with pancolitis from two studies of OPRM 2–4 g *versus* placebo (Hanauer *et al*.,^12^ PEN2A‐23_UC II^13^). Using a random effects model, which accounts for heterogeneity between studies by assuming that source data are not drawn from a common population (Microsoft Excel 365), OPRM resulted in significantly higher rates of complete or marked improvement in Physician's Global Assessment (absolute risk difference [ARD]: 22%; 95% CI: 7–37%) and significantly lower rates of treatment failure (ARD: 21%; 95% CI: 7–34%) compared to placebo. For patients with pancolitis, the PINCE study^14^, ^15^ reported significantly higher improvement rates (weeks 2, 4, and 8) and remission rate (week 8) with combined OPRM (4 g/day) and enema treatment (1 g/day) than oral therapy (4 g/day) alone.

## Conclusion

The new analyses presented here provide further evidence that OPRM is an effective treatment for the induction of remission in patients with moderately active UC.

## Data Availability

Data available from the corresponding author upon reasonable request.
